# Beyond femininity or masculinity: gender typologies and healthy eating in early adulthood

**DOI:** 10.1007/s00394-023-03268-9

**Published:** 2023-11-04

**Authors:** Juan Luis González-Pascual, Sara Esteban-Gonzalo, Óscar Luis Veiga, Laura Esteban-Gonzalo

**Affiliations:** 1 Department of Nursing and Nutrition, School of Biomedical and Health Sciences, Universidad Europea de Madrid, c/ Tajo s/n, Villaviciosa de Odón, 28670 Madrid, Spain; 2 Department of Personality, Evaluation and Psychological Treatment II, School of Psychology. Campus de Somosaguas, Universidad Complutense de Madrid, Pozuelo de Alarcón, 28223 Madrid, Spain; 3 Department of Psychology. School of Biomedical and Health Sciences, Universidad Europea de Madrid, c/ Tajo s/n, Villaviciosa de Odón, 28670 Madrid, Spain; 4 Department of Physical Education, Sport and Human Movement. School of Teacher Training and Education, Universidad Autónoma de Madrid, c/ Francisco Tomás y Valiente, 28049 Madrid, Spain; 5 Department of Nursing. School of Nursing, Physiotherapy and Podiatry, Universidad Complutense de Madrid, Plaza de Ramón y Cajal, 3, 28040 Madrid, Spain

**Keywords:** Gender, Gender typologies, Androgynous, Health, Healthy eating, Mediterranean diet

## Abstract

**Purpose:**

In the field of health sciences gender is often confused with biological sex (male/female) or reduced to a dichotomous classification (masculinity/femininity). The concepts of sex and gender interact with each other, but they are not equivalent. According to Sandra Bem four gender typologies can be established (androgynous, masculine, feminine and undifferentiated).

A relationship has been shown to exist between gender and health. Yet, there is little evidence as to the relationship between gender typologies and adherence to the Mediterranean diet. The aim of this research is to evaluate the association between Bem’s gender typologies and adherence to the Mediterranean diet.

**Methods:**

Mediterranean Diet Adherence Screener (MEDAS) and Bem’s gender typologies were the main variables. Sex, age, socioeconomic status (SES), body mass index (BMI) and obesity were analyzed as covariates.

**Results:**

Multilevel multivariate analysis showed that androgynous typology was associated with increased adherence to Mediterranean diet (*β* = 0.46 (SE 0.21), *p* = 0.033), adjusting by covariates, in a university population in Spain. Moreover, this was not the case with masculinity or femininity typologies.

**Conclusion:**

Thus, the results of this study suggest (1) that androgynous typology is not only associated with better mental health but also with healthy/healthier lifestyles, and (2) the complexity of the relationship between sex-gender and health would advise researchers avoid dichotomies such as male/female or masculinity/femininity.

## Introduction

The relationship between gender and health has been extensively studied and gender equity is recognized as a key factor in improving health equity by the World Health Organization [1]. Gender inequality puts women's health at risk and can be associated with other forms of discrimination based on age, race and socioeconomic status. Gender inequality puts women at greater risk of problems such as unwanted pregnancies, sexually transmitted infections, malnutrition and respiratory infections, among others [2].

However, studies into gender and health often confuse sex and gender when results are segmented between men and women [3, 4]. The concepts of sex and gender interact with each other, but they are not the same. Sex refers to biological and psychological characteristics of females and males. Gender refers to socially constructed characteristics of women and men [2, 3]. Sex and gender influence health differently [5].

A closer look at the concept of gender reveals that gender norms are “social norms defining acceptable and appropriate actions for women and men in a given group or society” [6]. According to the Focus Theory of Normative Conduct, norms can be prescriptive as standards to which people should conform [7]. In this sense, gender norms exist “in the mind” as beliefs about what is appropriate behavior for men and women, but they also exist “in the world”, embedded in institutions and reproduced in people's behaviors and roles [6].

How people perceive themselves or others in relation to gender norms can be expressed in terms of femininity (conforming to gender norms associated with women) or masculinity (conforming to gender norms associated with men) [3]. Conventional theories of masculinity-femininity assume that assessment of an individual's masculine and feminine qualities can be used to determine his or her position on a hypothetical masculinity-femininity continuum. However, nowadays it is believed that masculinity and femininity can be understood as two independent scales that can be measured in each person [3, 8]. According to Sandra Bem, the results of both scales can be combined to obtain four gender categorizations. People with low scores on both scales are undifferentiated, people with high scores on one of the scales are masculine or feminine, and people with high scores on both scales simultaneously are androgynous [9].

The relationship between gender norms and health has been studied to a lesser extent than the relationship between gender (or sex) and health. Conformity (or disconformity) with gender norms is associated with mental health [10–12], self-perceived health [13], and lifestyles related to noncommunicable diseases [12, 14, 15]. Furthermore, conformity (or disconformity) with gender norms in adolescents can impact their health outcomes as adults [16]. When analyzing conformity (or disconformity) with gender norms and health using Bem’s gender typology, results show that androgynous individuals present better results in mental health [17]. Since gender norms, unlike biological sex, are social conventions, conformity to them can be modified through a variety of interventions, including educational approaches. Gender norms, then, are relevant to the field of health and prevention [18].

Although research into the relation between gender and diet are scarce, some associations have been found in studies looking into lifestyles related to noncommunicable diseases [19]. The quantity and type of food preferred are deeply gendered and can be used as indicators of gender identity [20]. In general, women are more aware of the importance of healthy nutrition, show more interest in weight control, have a greater tendency to eat in stressful situations and are subject to greater social pressure for body image [21, 22]. On the other hand, men generally prefer more fatty foods with a strong taste and use fast food more frequently [21]. Therefore, masculinity is associated with a less healthy diet [23–25] characterized by high red meat consumption [26].

However, it seems that most studies focus only on men and women, or masculinity and femininity, and do not take Bem`s androgynous individuals into account. Only one study on Japanese workers found that androgyny is associated with higher healthy eating literacy [18]. But, according to Bem's model, androgynous individuals are characterized by their high adaptability and adherence to more adaptive standards and lifestyles, including those related to many aspects of health such as eating habits [27, 28]. With this in mind, we wondered to what extent these characteristics of androgyny could be generalized to eating habits.

The Mediterranean diet is recognized worldwide as one of the healthiest diets [29]. It is characterized by high intake of olive oil, vegetables particularly leafy green vegetables, legumes, cereals, fruits and nuts, moderate intakes of fish/seafood, dairy products and wine, and low intakes of red meat, eggs and sweets [30]. This diet is associated with multiple beneficial health effects such as lower risk of cardiovascular disease and cancer [29, 31, 32], as well as better mental health [33, 34].

Considering the not yet well-known link between gender and diet, and, to the best of our knowledge, the lack of previous studies in this regard, the objective of this study was to assess the relationship between gender norms and adherence to the Mediterranean diet (as a healthy dietary pattern). Furthermore, since most research focuses only on masculinity and femininity, it seems appropriate to broaden the focus to include other gender typologies, like the androgynous group as well. Finally, the study´s authors determined appropriate to conduct this study with early adults as subjects (university population) since gender norms in youth influence health outcomes in adulthood.

## Methods

### Study design

The present cross-sectional study was designed to value the associations between gender typologies and adherence to Mediterranean diet.

### Participants and setting

Participants were recruited from three universities in Spain, two in Madrid and one in Toledo. The inclusion criteria were to be enrolled in any university course and to be able to fill in the questionnaire in Spanish language.

Participants were from several academic fields of study (Health Sciences Vocational Training Cycles, Undergraduate studies: Psychology, Nursing, Medicine, Odontology, Education, Physical Education, Physiotherapy and Criminology, Master`s degree: Education Physical Education and Health Sciences, and PhD students) through a convenience sampling.

Data were collected from February to November 2019 during one of the classes in which the students were enrolled. A total of 775 students provided valid data.

### Measurement instruments

#### Healthy eating

The Spanish version of the 14-point Mediterranean Diet Adherence Screener (MEDAS) was used to measure adherence to Mediterranean diet as a healthy eating pattern. MEDAS was developed in Spain [35] and has been used to measure adherence to the Mediterranean diet in various countries [36] and age groups, including university students [37]. Participants rated themselves on 14 items: 12 questions on food consumption frequency and 2 questions on food intake habits considered characteristic of the Mediterranean diet. Each question was scored 0 or 1. The total score rates from 0 to 14 points. The higher the rate, the more adherence to Mediterranean diet.

#### Gender typologies

The Bem Sex–Role Inventory (BSRI) was used to measure gender perceptions and their behavioral and attitudinal correlates [9].

The BSRI is a self-descriptive questionnaire that, in its original English version, presents a list of 60 adjectives, of which 20 correspond to cultural stereotypes of feminine traits (e.g., affectionate, sensitive to the needs of others, tender, fond of children), 20 correspond to stereotypically masculine traits (e.g., ambitious, independent, energetic, assertive), and the remaining 20 are neutral (e.g., happy, moody, reliable, jealous) [38]. The 20 neutral items are to prevent response bias, ten of them refer to attributes that are socially considered desirable (e.g., adaptable, honest, formal) and the remaining ten to undesirable ones (e.g., inefficient, dramatic, jealous) [39].

The original 60-items version has been translated and validated in Spanish with acceptable internal consistency (Cronbach's alpha for the femininity traits was 0.75 in women and 0.83 in men, and for the masculinity traits, it was 0.79 in women and 0.80 in men) [38]. In a subsequent review with university students, the results regarding internal consistency were confirmed (Cronbach's alpha for the femininity traits was 0.79 in women and 0.81 in men, and for the masculinity traits, it was 0.85 in women and 0.82 in men) [39].

Participants were required to respond on a 7-point Likert scale (from (from never/almost never to always/almost always) to indicate the degree of self-identification with each of the 60 personality traits presented.

For the interpretation of the scale only the score of the feminine and masculine traits are considered, not the neutral ones. Based on the score, the BSRI offers four different possible resulting typologies: masculine (high score on masculine traits, low score on feminine traits), feminine (high score on feminine traits, low score on masculine traits), androgynous (high scores on both feminine and masculine traits simultaneously), and undifferentiated (low scores on both feminine and masculine traits) [9, 38–40]. The inventory has been found to satisfactorily measure the impact of gender roles on health outcomes, justifying its use in epidemiological studies [41].

#### Covariates

Covariates were included to adjust for confounding variables that could be associated with healthy eating and gender typologies. The potential covariates considered were sex, age, socioeconomic status (SES), body mass index (BMI) and obesity. All the covariates were self-reported.

Sex was collected as biological sex at birth (male / female) and age was collected in years. Self-reported weight and height were collected, and body mass index was calculated as weight (in kilograms) divided by the square of height (in meters). Obesity status was defined as body mass index equal or greater than 30.

Socioeconomic status (SES) was defined according to level of education (no studies, primary school, secondary school, high school, technical education degree, university). The score rates from 0 (no studies) to 5 (university). In those participants who were economically dependent, the average between student’s mother and father’s level of education was considered. In participants who were financially independent, their own level of education was considered.

### Data analyses

Descriptive statistics were performed using relative frequencies in the case of qualitative variables and mean and standard deviation in the case of quantitative variables. Bivariate analysis was performed between adherence to the Mediterranean diet (MEDAS) and the quantitative variables using the Pearson correlation coefficient. Bivariate analysis was carried out between adherence to the Mediterranean diet (MEDAS) and qualitative variables using the T-Student test or ANOVA depending on whether they were dichotomous or polychotomous variables. In addition, bivariate analysis was performed between the results of the questions that make up the MEDAS questionnaire and the gender typologies using the chi-square test. Bilateral differences were considered significant at *p* < 0.05.

Finally, multivariate analysis was performed using multilevel mixed-effects linear regression. The models were constructed by introducing the dependent variable adherence to Mediterranean diet and, separately, each of the independent variables of the gender typology categorizations. In addition, the covariates were introduced one by one into each model. Covariates that did not show an association with adherence to Mediterranean diet or modify the significance of the gender typology were excluded from the final model. All models included a random intercept for university, university study, and class. This multilevel approach was selected due to the existence of different levels of grouping of the study participants, which level of association should be considered when performing the regression models. Unadjusted models and adjusted models were fitted. The β value, the β Standard Error (SE) and the β 95% Confidence Interval (CI) were calculated. The statistical package IBM SPSS version 25 was used.

## Results

A total of 775 students (236 men) provided valid data. The characteristics of participating students are shown in Table 1. Most were females (69.6%), the mean age was 22.6 years old, adherence to Mediterranean diet was 6.4 out of 14 and their gender typologies were 32.1% feminine, 18.3% masculine, 18.2% androgynous and 31.4% undifferentiated.

**Table 1:** Characteristics of the participants (*n* = 775)

Age^a^	22.6 (5.6)
Sex^b^	
- Male	30.4%
- Female	69.6%
Socioeconomic status (SES) (0–5)^a^	4.0 (1.0)
Body mass index (BMI)^a^	22.3 (3.3)
Obesity^b^	
- Yes	2.6%
- No	97.4%
Gender typologies (BSRI)^b^	
- Feminine	32.1%
- Masculine	18.3%
- Androgynous	18.2%
- Undifferentiated	31.4%
Adherence to Mediterranean diet (MEDAS) (0–14)^a^	6.4 (2.0)

^a^Mean (standard deviation)

^b^Relative frequencies (%)

The bivariate analysis of the relationship between adherence to Mediterranean diet and gender typologies as well as the covariates are shown in Table 2.

**Table 2:** Relationship between adherence to Mediterranean diet and variables (*n* = 775)

	Adherence to Mediterranean diet (MEDAS) (0–14)	*p* value
Sex^a^		
Male (*n* = 236)	6.3 (2.0)	0.612
Female (*n* = 535)	6.4 (1.9)	
Age^b^	0.187	**< 0.001**
Socioeconomic status (SES)^b^	0.059	0.115
Body mass index (BMI)^b^	− 0.005	0.891
Obesity^a^		
Yes (*n* = 21)	5.7 (1.7)	0.107
No (*n* = 753)	6.4 (2.0)	
Gender typologies^c^		
Feminine (*n* = 199)	6.2 (1.9)	**0.003**
Masculine (*n* = 117)	6.6 (2.0)	
Androgynous (*n* = 115)	6.8 (2.1)	
Undifferentiated (*n* = 196)	6.0 (1.9)	

Values are median (standard deviation) or correlation coefficient. Statistically significant values are in bold type (*p* < 0.05)

^a^*T*-Student

^b^Pearson correlation

^c^ANOVA

Adherence to Mediterranean diet was associated with gender typology (*p* = 0.003) and age (*p* < 0.001).

In general, no statistically significant differences were found at the level of the individual questions that make up the MEDAS questionnaire according to gender typologies. Differences were only found in adherence to the recommended consumption of nuts (Feminine 14.5%, Masculine 28.8%, Androgynous 21.4%, Undifferentiated 15.3%; *p* = 0.006) and desserts (Feminine 55.4%, Masculine 70.3%, Androgynous 72.6%, Undifferentiated 58.8%; *p* = 0.013).

Multilevel mixed-effects linear regression models for the associations between adherence to Mediterranean diet and gender typologies are shown in Table 3. There were no relevant variations between unadjusted and adjusted models, thus results refer to the final adjusted model. Androgynous typology was associated with increased adherence to Mediterranean diet (*β* = 0.46 (SE 0.21), *p* = 0.033), adjusting for age. Feminine, masculine and undifferentiated typologies did not show statistical association with Mediterranean diet.

**Table 3:** Linear regression models for the association between adherence to Mediterranean diet and Gender typologies

Gender typologies	Unadjusted model			Adjusted model 1^a^			Final adjusted model^b^		
Covariates	*β* (SE)	*β* 95% CI	*p* value	*β* (SE)	*β* 95% CI	*p* value	*β* (SE)	*β* 95% CI	*p* value
Feminine	− 0.23 (0.18)	− 0.57–0.12	0.205						
Masculine	0.29 (0.21)	− 0.12–0.71	0.166						
Androgynous	**0.53 (0.22)**	**0.10–0.95**	**0.015**	**0.50 (0.23)**	**0.06–0.95**	**0.026**	**0.46 (0.21)**	**0.04–0.88**	**0.033**
Age				**0.08 (0.02)**	**0.05–0.11**	**< 0.001**	**0.08 (0.02)**	**0.04–0.11**	**< 0.001**
Sex				0.21 (0.19)	− 0.16–0.58	0.266			**0.033**
SES				0.09 (0.08)	− 0.07–0.25	0.292			
BMI				− 0.00 (0.03)	− 0.06–0.06	0.961			
Obesity				− 1.01 (0.59)	− 2.16–0.14	0.086			
Undifferentiated	− 0.35 (0.18)	− 0.70–0.01	0.055						

Statistically significant values are in bold type (*p* < 0.05)

^a^Adjusted model 1, analyses were adjusted for age, sex, socioeconomic status (SES), body mass index (BMI) and obesity

^b^Final adjusted model, analyses were adjusted for age

## Discussion

The objective of this research was to value the associations between adherence to the Mediterranean diet as a healthy dietary pattern and gender typologies.

Overall, adherence to the Mediterranean diet was reasonable, which is consistent with research published for adolescents in Europe [42] and university students in Spain [37].

Older age was associated with greater adherence to the Mediterranean diet. This result is coherent with previously published research which has shown that adherence to Mediterranean diet, although relatively stable during adulthood, tends to show some improvement over time [43].

Although we found few significant differences at the level of the individual questions that make up the MEDAS questionnaire in relation to gender typologies, the differences arise considering the overall result of the questionnaire. Androgynous typology was associated with increased adherence to Mediterranean diet, even adjusting by other covariates, including age. However, feminine, masculine and undifferentiated typologies showed no association with adherence to Mediterranean diet. These results are consistent with those obtained in a study in Japan, where androgynous typology reported greater self-efficacy for healthy eating among Japanese in early adulthood [18]. Also, previous ideas regarding the adaptive role of androgyny have emerged. Androgynous individuals appear, once again, to be more effective and adaptive when facing and resolving health demands by using more efficient coping strategies [27, 28]. Our results allow us to extend these coping strategies to eating habits and healthy eating styles.

No association has been found between participants´ biological sex and adherence to the Mediterranean diet which is consistent with other results found in university students in Spain [37]. This highlights the complex relationship between sex and gender. As occurs in many other issues, disaggregating results of adherence to Mediterranean diet by sex does not yield the same results as by gender typology [3–5]. This study, as well as another carried out recently [11], point out the need to consider gender, rather than sex, the most accurate predictor of health orientation and health-related behaviors.

Moreover, the dichotomy femininity/masculinity does not encompass all the gradations that can be presented when analyzing gender. For instance, our results show that feminine and masculine typologies are not associated with adherence to the Mediterranean diet by themselves, although the androgynous typology is.

Since androgynous typology is characterized by high scores in both masculinity and femininity, these results seem contradictory to studies associating masculinity with a less healthy diet [23–25].

One possible explanation may be that alcohol consumption is associated with masculinity [44, 45], and daily wine consumption is one of the hallmarks of the Mediterranean diet [30]. Another explanation could be related to new trends in masculinity in that hybrid masculinities maintain typical characteristics of traditional masculinity, while selectively adopting some characteristics related to femininity or subordinate masculinities [46]. New masculinities have been found to allow for healthier diets [47, 48].

Similarly, these results also seem contradictory with studies that associate femininity with a healthier diet [15, 21]. Although on the one hand high scores in femininity contribute to androgynous typology, female typology is not associated with greater adherence to Mediterranean diet in our results. One possible explanation would be that femininity is not only associated with healthier diet, but with greater body image dissatisfaction, desire for thinness and, in extreme cases, eating disorders [49]. Thus, the predisposition to follow a healthy diet could be counterbalanced by desire for thinness, leading to lower adherence to the Mediterranean diet than people of androgynous gender typology.

Androgynous typology is related to people who experiment little social pressure to conform to gender stereotypes prevalent in their culture, so they are able to base their actions on other reasons rather than on gender norms. By not being restricted by gender norms, they have more behavioral options [50]. In this sense, people of androgynous typology may feel free to adhere more strongly to the Mediterranean diet, taking advantage of the best of factors associated with femininity and masculinity. One could subsequently consider that androgynous individuals are endowed with less rigid and more flexible behavioral patterns, and that these patterns could be applicable to more areas of health than those known today.

This study is, as far as we know, the first to find an association between androgynous gender typology and adherence to Mediterranean diet, controlling for other variables, and independently of sex. Its strengths are sample size and multi-level multivariate statistical analysis. The main limitations have to do with it being a cross-sectional study, so the association between variables does not imply a cause-effect relationship. In addition, although validated instruments have been used, the data are self-referenced.

## Conclusion

Androgynous typology was associated with increased adherence to Mediterranean diet, even adjusting for other covariates. Feminine, masculine and undifferentiated typologies did not show association with adherence to Mediterranean diet. The study highlights the role of androgyny as predictor of flexible and adaptative healthy habits that allow individuals to better cope with health demands, on this occasion in relation to health-related eating behaviors.

No association was found between biological sex of participants and adherence to Mediterranean diet. The study also highlights the need to go beyond dichotomies (male / female, masculinity / femininity) when studying the relationship between gender and health.

## Data Availability

The data that support the findings of this study are available from the corresponding author, upon reasonable request by email.
